# Zika Virus as Oncolytic Therapy for Brain Cancer: Myth or Reality?

**DOI:** 10.3389/fmicb.2019.02715

**Published:** 2019-11-20

**Authors:** Kar Yan Su, Vinod R. M. T. Balasubramaniam

**Affiliations:** ^1^Jeffrey Cheah School of Medicine and Health Sciences, Monash University Malaysia, Bandar Sunway, Malaysia; ^2^School of Science, Monash University Malaysia, Bandar Sunway, Malaysia

**Keywords:** Zika, neurotropism, glioblastoma, MSI1, AXL, oncolytic

## Abstract

The ability of self-replicating oncolytic viruses (OVs) to preferentially infect and lyse cancer cells while stimulating anti-tumor immunity of the host strongly indicates its value as a new field of cancer therapeutics to be further explored. The emergence of Zika virus (ZIKV) as a global health threat due to its recent outbreak in Brazil has caught the attention of the scientific community and led to the discovery of its oncolytic potential for the treatment of glioblastoma multiforme (GBM), the most common and fatal brain tumor with poor prognosis. Herein, we evaluate the neurotropism of ZIKV relative to the receptor tyrosine kinase AXL and its ligand Gas6 in viral entry and the RNA-binding protein Musashi-1 (MSI1) in replication which are also overexpressed in GBM, suggesting its potential for specific targeting of the tumor. Additionally, this review discusses genetic modifications performed to enhance safety and efficacy of ZIKV as well as speculates future directions for the OV therapy.

## Introduction

Oncolytic viruses (OVs) are viruses that are naturally cancer-selective or can be genetically modified to reduce pathogenicity, increase lytic potential, as well as induce innate and adaptive anti-tumor immunity of the host. OV therapy has unique advantages that can overcome the shortcomings of current therapies. Preclinical models and clinical trials conducted have provided evidence for the effectiveness of OVs in treating cancer. These viruses preferentially infect and kill cancer cells because antiviral defense responses such as interferons (IFNs) found in normal cells are usually inactivated as part of the malignant phenotype. Therefore, the specificity of OV therapy toward cancer cells allows minimal side effects. The ability to self-replicate in the tumor means that lower dose of OV is required and the viral dosage amplifies in the tumor with time to infect nearby tumor cells, which is in contrast with conventional drug pharmacokinetics that reduces with time (Chiocca and Rabkin, 2014). Moreover, the chances of generating resistance against OVs are low and resistance has not been seen so far. This is because OVs usually target numerous oncogenic pathways and utilize many means for cytotoxicity (Chiocca and Rabkin, 2014). OV therapy is also a type of immunotherapy whereby the virus activates the local innate immune response and the tumor-associated antigens released by lysed tumor cells primes the systemic adaptive anti-tumor immunity capable of targeting uninfected cancer cells at distant, uninjected sites.

There are many types of viruses with oncolytic potential that are yet to be discovered. Zika virus (ZIKV) has recently gained the attention of the public and has been declared as a public health emergency of international concern in 1 February 2016 by the World Health Organization (WHO) (WHO, 2017) due to the outbreak in Brazil, which has caused congenital abnormalities such as microcephaly and neurological disorders including Guillain-Barré syndrome. The virus has been shown to utilize the receptor tyrosine kinase AXL and its ligand Gas6 for viral entry (Meertens et al., 2017), albeit contradictory findings on the role of the receptor in ZIKV infection were reported (Wells et al., 2016; Hastings et al., 2017; Li et al., 2017; Wang et al., 2017). Additionally, the RNA-binding protein Musashi-1 (MSI1) was found to be required for the viral replication (Chavali et al., 2017). Interestingly, these crucial proteins implicated with ZIKV infection in humans are also overexpressed in cancers, including glioblastoma multiforme (GBM), the most common and lethal malignant brain tumor with poor prognosis. Despite aggressive treatment with radiation and chemotherapy, most patients have a poor median survival of below 2 years (Stupp et al., 2009). Diffuse migration and local invasion of cancer cells into adjacent tissues, which shield them from surgery and radiation, are the hallmarks of aggressive GBM (Hou et al., 2006). The presence of treatment-resistant glioblastoma stem cells (GSCs) is considered as a main cause of tumor recurrence. Importantly, ZIKV was shown to exhibit oncolytic activity against GSCs (Zhu et al., 2017) and a recent study have demonstrated the treatment of human GBM with a live attenuated ZIKV vaccine candidate which targets GSCs (Chen et al., 2018). Herewith, we analyze the potential of ZIKV as an OV by exploiting its neurotropism and the genetic modifications performed to improve safety and efficacy against GBM. In addition, this review speculates future directions for ZIKV as an OV therapy.

## Glioblastoma Multiforme

Each year, approximately five to six cases from 100,000 individuals are diagnosed with primary malignant brain tumors, whereby about 80% are malignant gliomas (Schwartzbaum et al., 2006; Stupp et al., 2010). GBM accounts for greater than half of the malignant glioma cases and is correlated with high mortality and morbidity, despite current treatment efforts (Alifieris and Trafalis, 2015). Clinically, GBM patients may present with headaches, confusion, seizures, memory loss, personality changes, or focal neurologic deficits. GBM clinical subtypes are described as primary GBM, the most common form which arises *de novo* in older patients, and secondary GBM that occurs from prior low grade astrocytomas in younger patients (Kleihues and Ohgaki, 1999). Primary GBM usually has mutated, amplified epidermal-growth factor receptor (EGFR) while secondary GBM has enhanced signaling via PDGF-A receptor. Both types of mutations result in amplified tyrosine kinase receptor (TKR) activity and subsequently activation of PI3K and RAS pathways. Lastly, development of low-grade glioma to high-grade involves inactivation of retinoblastoma gene (RB1) and enhanced activity of human double minute 2 (HDM2) (Kleihues and Ohgaki, 1999; Louis, 2006). These abnormalities cause aberrant regulation of cell cycle and growth-factor-mediated signaling pathways as well as involved with inhibition of apoptosis, enhanced cell proliferation, invasion, and angiogenesis (Louis, 2006). GBM has also been classified by the Cancer Genome Atlas (TCGA) into molecular subtypes, namely classical, mesenchymal, proneural, and neural classes based upon genetic changes and expression profiles. In fact, primary and secondary GBM may be histologically indistinguishable but vary in genetic and epigenetic profiles. Furthermore, GSCs contribute to tumor malignancy through sustained proliferation, immune escape, enhanced angiogenesis, invasive potential, and therapeutic resistance (Bao et al., 2006; Alvarado et al., 2017). Intriguingly, recent studies have revealed oncolytic activity of ZIKV against GSCs (Zhu et al., 2017) and treatment of human GBM with a live attenuated ZIKV vaccine candidate targeting these cells has been demonstrated (Chen et al., 2018).

## Zika Virus

Zika virus was initially isolated in 1947 from a sentinel rhesus monkey in the Zika forest of Uganda, followed by a second isolation from the mosquito *Aedes africanus* at the same site in the next year (Dick et al., 1952). This arbovirus is mainly transmitted to humans via the bite of blood-feeding female *A. aegypti* (Garcia et al., 1969; Elshahawi et al., 2019) mosquitoes, besides other *Aedes* spp., namely *A. albopictus* (Grard et al., 2014), *A. hensilli* (Duffy et al., 2009; Ledermann et al., 2014), *A. africanus* (Haddow et al., 1964), and *A. luteocephalus* (Lee and Moore, 1972). Most cases of the infection were asymptomatic and around 20% of infected individuals show mild symptoms that resemble those caused by other arboviruses, such as dengue or chikungunya viruses. Hence, ZIKV infection was thought to cause a self-limiting and mild febrile disease until the epidemic outbreaks on Yap Island and Guam, Micronesia in 2007, followed by French Polynesia in 2013–2014 as well as Brazil and other Latin American countries in 2015–2016. The number of cases has increased significantly and the infection was associated with microcephaly and other congenital abnormalities in fetus of infected mothers (Brasil et al., 2016a) as well as Guillain-Barré syndrome (Brasil et al., 2016b; Cao-Lormeau et al., 2016; Watrin et al., 2016), meningoencephalitis (Carteaux et al., 2016), myelitis, and ophthalmologic abnormalities in infected adults (Araujo et al., 2016). Other modes of transmission have also been discovered such as blood transfusion as well as sexual (Foy et al., 2011; Musso et al., 2015b), *in utero* (Brasil et al., 2016a), and perinatal transmission (Besnard et al., 2014) with evidence of ZIKV being found in serum (Besnard et al., 2014; Brasil et al., 2016a), urine (Gourinat et al., 2015; Brasil et al., 2016a), saliva (Musso et al., 2015a), semen (Musso et al., 2015b), cerebrospinal fluid (de Fatima Vasco Aragao et al., 2016; Hazin et al., 2016), amniotic fluid (Calvet et al., 2016), breast milk (Dupont-Rouzeyrol et al., 2016) as well as vaginal (Nicastri et al., 2016), cervical (Prisant et al., 2016), and rectal (Bôtto-Menezes et al., 2019) secretions.

Zika virus is a member of the *Flavivirus* genus of Flaviviridae family where dengue, yellow fever, West Nile, Japanese encephalitis, and tick-borne encephalitis viruses are among the human pathogens belonging to the same family. It is believed that ZIKV has only one serotype with two geographically distinct lineages, African and Asian, through phylogenetic analyses (Haddow et al., 2012;Tham et al., 2018). This 50 nm virion is a single-stranded, positive-sense RNA virus. Its 10.7 kb genome contains two flanking untranslated regions (5′- and 3′-UTRs) and a long open-reading frame encoding a polyprotein that is cleaved into three structural proteins [capsid (C), premembrane/membrane (prM/M), and envelope (E)] as well as seven non-structural proteins (NS1, NS2A, NS2B, NS3, NS4A, NS4B, and NS5) using host (unknown cellular signalase) and viral (NS3 serine protease) proteases (Lindenbach and Rice, 2003; Kuno and Chang, 2007). ZIKV contains a nucleocapsid that is enclosed by a lipid bilayer consisting of the prM/M and E proteins, which are arranged in icosahedral symmetry on the surface (Kostyuchenko et al., 2016; Sirohi et al., 2016). Flavivirus envelope protein serves as a receptor binding and membrane fusion protein. It is also the main target of neutralizing antibodies from the host. During virion assembly, the complex formed by the membrane protein protects the envelop protein from degradation. Meanwhile, non-structural proteins are implicated in viral replication and inhibition of cellular innate immune responses (Lindenbach and Rice, 2003).

## CNS Invasion by ZIKV

In order for ZIKV to target GBM in an OV therapy, it would be necessary for the virus to cross the blood–brain barrier and invade the CNS. ZIKV has been detected in CSF of encephalitic adult as well as in post-mortem brains of microcephalic fetuses and stillborns (Carteaux et al., 2016; Mlakar et al., 2016) signifying CNS invasion by the virus. Indeed, these findings are consistent with the numerous studies which showed that ZIKV is neurotropic. Nonetheless, the mechanisms of brain invasion are yet to be resolved. Recent evidences have suggested that endothelial cells from the blood–brain barrier are permissive to replication of various ZIKV strains and that the virus could cross the endothelial barrier, without causing significant increase in its permeability (Bramley et al., 2017; Mladinich et al., 2017; Papa et al., 2017; Alimonti et al., 2018). Papa et al. (2017) suggested that ZIKV may cross the endothelial barrier via transcytosis or via endocytosis and exocytosis replication pathways. In addition, mouse models from the study also substantiated the hypothesis whereby ZIKV might cross the blood–brain barrier without severe disruption. Intriguingly, barrier breakdown was observed at later time points following the infection. Nevertheless, it is unclear whether the barrier breakdown was crucial for the neurological diseases or it was a consequence of a severe disease. Notably, there are large differences between ZIKV strains which led to dissimilar findings associated with the viral infections (Papa et al., 2017). Thus, further investigations on the different ZIKV isolates may clarify the mechanisms involved in disease.

## AXL in ZIKV Entry

Zika virus penetrates host cells through receptor-mediated endocytosis. Viral entry is mediated by envelope proteins that interact with attachment factors or cell surface receptors in which their differential expression determines the cellular tropism of the virus. AXL from the TYRO3, AXL, and MERTK (TAM) family of receptor tyrosine kinases was recently found to support ZIKV infection of human glial cells (Meertens et al., 2017), neural stem cells (Nowakowski et al., 2016; Onorati et al., 2016), skin fibroblasts (Hamel et al., 2015), and fetal endothelial cells (Richard et al., 2017). Studies have shown that AXL which regulates inflammatory processes mediates ZIKV entry in human glial cells and dampens the innate immune responses (Meertens et al., 2017). The receptor binds to ZIKV through growth arrest-specific 6 (Gas6) which acts as a cofactor to promote viral infection. Both AXL and its ligand Gas6 are essential for ZIKV infection and blocking either one of them would inhibit the infection (Meertens et al., 2017). The ZIKV–Gas6 complex activates AXL kinase activity during viral entry to down modulate type 1 IFN signaling and facilitate infection. The binding is followed by ZIKV internalization via clathrin-mediated endocytosis and traffics to Rab5^+^ endosomes to establish the infection (Meertens et al., 2017). Noteworthily, besides serving as an important entry factor for ZIKV infection, AXL and its ligand Gas6 also play an essential role in diverse types of cancer, including GBM which originates from glial cells (Hutterer et al., 2008). As such, the overexpression of AXL and Gas6 in GBM would potentially allow the specific targeting of ZIKV in OV therapy. Indeed, a recent study has demonstrated the oncolytic activity of ZIKV against GSCs (Zhu et al., 2017). Nevertheless, the role of AXL in ZIKV infection is still controversial as contradictory findings were reported. Recent contrasting findings indicated that AXL is not needed in ZIKV infection in human neural progenitor cells, cerebral organoids (Wells et al., 2016), and mouse models (Hastings et al., 2017; Li et al., 2017; Wang et al., 2017). Hence, the exact mechanism underlying the entry of ZIKV into the host cells remains unclear. Although the *bona fide* entry receptors remain unresolved, many cell surface molecules have shown to contribute to the infection such as the C-type lectins DC-SIGN and L-SIGN as well as phosphatidylserine receptors including members of T-cell Ig mucin (TIM) family, besides the TAM family (Meertens et al., 2012). Consequently, further investigations are warranted to study the mechanism of ZIKV infection.

## MSI1 in ZIKV Replication

The 3′-UTR of ZIKV genome interacts with the neural RNA-binding protein MSI1 for viral replication in both primary and transformed neural cell lines (Chavali et al., 2017). MSI1 promotes ZIKV UTR-driven translation and may stabilize its genome as well as regulate its synthesis or cyclization, given that host RNA-binding proteins are known to interact with UTRs to mediate translation, stabilization, and replication of viral genome. MSI1 also exerts pro-proliferative effects such as cyclin-dependent kinase activity which are required for ZIKV replication (Xu et al., 2016). Notably, MSI1 expression decreases with differentiation. The protein was found to be highly enriched in neural precursor cells of the human embryonic brain, but not in mature neurons (Chavali et al., 2017). Thus, this could explain why ZIKV primarily infects the fetal brain when compared to the adult brain which consists of more mature neurons (Zhu et al., 2017). Additionally, MSI1 is also largely expressed in spermatogonia and mitotic gonocytes, suggesting the reason for the presence of ZIKV in semen (Foy et al., 2011; Musso et al., 2015b) of infected individuals which could result in sexual transmission of the virus.

The abundant ZIKV RNA present in infected cells competes for available MSI1 with its endogenous targets, including the mRNA of microcephalin (MCPH1) which is expressed in the developing fetal brain. This decreases MSI1 interaction with its normal targets and leads to changes in the protein levels of MCPH1 as MSI1 plays a role in translational activation of the protein (Chavali et al., 2017). MSI1 regulates chromosome condensation by controlling the expression of MCPH1 and reducing the expression has led to an increase in the frequency of premature chromosome condensation (PCC) during cell division (Chavali et al., 2017). Noteworthily, impaired chromosome condensation has been recently found to cause microcephaly (Martin et al., 2016). Indeed, MSI1 is necessary in neurodevelopment for both vertebrates and invertebrates in which microcephaly is exhibited by MSI1-depleted zebrafish (Shibata et al., 2012) and mutant mice displaying thin cerebral cortex besides other morphological brain abnormalities (Sakakibara et al., 2002). Individuals with mutated MSI1 also exhibited autosomal recessive primary microcephaly (Chavali et al., 2017).

## The Role of MSI1 in GBM

The crucial function of MSI1 in the maintenance of stem cell self-renewal also suggests its potential role in oncogenesis when dysregulated or aberrantly reactivated as the undifferentiated stem cell state is an indispensable feature of cancer. Evidently, overexpression of MSI1 has been observed in several types of tumors, including GBM (Toda et al., 2001), and is regarded as a well-established marker for tumor metastasis and recurrence. MSI1 expression was found to be correlated with the proliferative activity and grade of malignancy in glioma (Toda et al., 2001), which is the most common form of central nervous system (CNS) tumor that derives from glial cells. Tumors with strong MSI1 expression have high proliferative activity. Indeed, GBM, the most malignant form of glioma, exhibited greater MSI1 expression compared with less malignant gliomas and non-neoplastic brain tissue (Toda et al., 2001). MSI1 overexpression was reported to alter morphology, enhance migration, as well as increase viscoelasticity and flexibility of GBM cells (Chen et al., 2017). Furthermore, the overexpression was shown to down-regulate pro-apoptotic genes and protect GBM cells from drug-induced apoptosis (Chen et al., 2016).

Although studies have shown that targeting MSI1 inhibits cancer growth (Lan et al., 2015), drugging the protein to inhibit its function in cancers remains a challenge as RNA-binding proteins are not enzymes, which have traditional catalytic pockets for inhibition. This warrant alternative approaches in targeting the protein for cancer treatment. Remarkably, the unique neurotropism of ZIKV provides an alternative strategy for the treatment of GBM in adults given the overexpression of MSI1 (Toda et al., 2001) as well as AXL and its ligand Gas6 which are important for the viral infection. The high expression level of MSI1 needed for ZIKV replication in the tumors also indicates that the virus would be able to self-replicate, which is an important feature of effective OV therapy. Moreover, the negligible MSI1 expression in most adult tissues and the presence of antiviral defense responses, such as IFN which restricts virus replication in normal cells, suggest high specificity of ZIKV infection toward the tumor with limiting side effect in patients. Indeed, ZIKV preferentially infected and killed GSCs compared to normal neuronal cells or differentiated tumor progeny (Zhu et al., 2017). ZIKV preference of infecting GSCs may be due to the presence of higher levels of MSI1 in relative to the differentiated glioma cells (DGCs), giving rise to the stem-like, self-renewing, tumorigenic properties of these tumor-initiating cells (Chen et al., 2012). Thus, the essential role of MSI1 further supports the rational of utilizing ZIKV in OV therapy for GBM. Moreover, GBM rarely metastasize out of the CNS and often recurs in proximity to the original resection cavity (Wallner et al., 1989). Hence, local therapies such as OV therapy have been investigated (Alonso et al., 2012; Cassady et al., 2017).

## Genetic Modifications of ZIKV in OV Therapy

To further optimize the safety and efficacy of ZIKV for OV therapy, the virus has been genetically modified. NS5 of ZIKV has been shown to impede type I IFN induction and signaling (Hertzog et al., 2018). ZIKV antagonizes type I IFN response through NS5 by promoting proteasomal degradation of the transcription factor STAT2 (Grant et al., 2016; Kumar et al., 2016), which is activated downstream of signaling by the type I IFN receptor (Ifnar1). Mutation in the *Flavivirus* NS5 gene causes translational inhibition of the virus by type I IFN and IFN-induced proteins with tetratricopeptide repeats (IFIT) leading to attenuation of viral replication in cells responsive to type I IFN (Daffis et al., 2010). Furthermore, attenuated ZIKV with the mutation was shown to maintain effectiveness against GSCs and has additive effects with temozolomide chemotherapy (Zhu et al., 2017), which is often resisted by GSCs (Stupp et al., 2009; Chen et al., 2012). The mutant ZIKV has two nucleotide changes in the same codon of NS5 gene and regression to pathogenic virus requires a low-probability event of concurrent nucleotide changes to produce the exact amino acid reversion (Zhu et al., 2017). Moreover, the virus could be further sensitized to type I IFN by creating second-site mutations at the 3′-UTR which affect the production of a sub-genomic RNA (Akiyama et al., 2016; Donald et al., 2016). These studies suggest that engineered mutant ZIKV strains may enhance infection and lysis of GSCs with lower toxicity to neighboring differentiated neural cells. In fact, a live attenuated ZIKV vaccine candidate with a 10-nucleotide deletion in the 3′-UTR was recently shown to substantially reduce intracerebral tumor growth and prolonged animal survival by specifically killing GSCs within the tumor (Chen et al., 2018). The virus infection stimulated antiviral immunity, inflammation, and apoptosis of GSCs with excellent safety profile upon intracerebral injection into the mice (Chen et al., 2018).

## Future Directions

Although recent studies demonstrated the treatment of GBM using attenuated ZIKV, which primarily targets GSCs, the main cause of tumor recurrence and resistance, both the wild-type and mutant viruses have lower effect on DGCs which are also an important constituent of GBM (Zhu et al., 2017; Chen et al., 2018). IFN signaling is a determinant of differential sensitivity of GSCs toward ZIKV as type I IFN-stimulated genes (ISGs), which cannot be fully antagonized by the virus, were shown to be upregulated in DGCs (Zhu et al., 2017). Hence, IFN responses may contribute to the preference of ZIKV in targeting GSCs compared to the limited killing of DGCs and normal neural cells (Zhu et al., 2017). Thus, this raises the question of how we can effectively target DGCs using ZIKV in OV therapy. One remarkable advantage of using OV therapy in cancer treatment is that these viruses are known to stimulate anti-tumor immunity of the host as they lyse the cancer cells and release IFNs, chemokines, Toll-like receptor agonists, tumor-associated antigens, danger-associated molecular patterns (DAMPs), and pathogen-associated molecular patterns (PAMPs). These components change the immunosuppressive tumor microenvironment to a more pro-immunogenic environment for adaptive immune priming, leading to a long-term vaccination effect. Therefore, can the anti-tumor immunity stimulated by the attenuated ZIKV strains target the DGCs or even metastatic tumors at distant sites? However, currently, the anti-tumor immunity or systemic efficacy has not been clearly addressed in the recent studies demonstrating oncolytic properties of ZIKV (Zhu et al., 2017; Chen et al., 2018) and further investigations are required to better understand how OVs regulate immune responses in patients. As such, there remains a research gap in which the anti-tumor immunity of genetically modified ZIKV strains are yet to be elucidated. Besides that, it is worthy to note that more fundamental researches are required to learn more about ZIKV biology in order to better utilize the virus in OV therapy.

Zika virus can be engineered to induce systemic anti-tumor immunologic responses at distant, uninjected sites including DGCs which are less infected by ZIKV, by arming the virus with transgenes expressing immunomodulatory proteins, such as antibodies, chemokines, interleukins, or IFNs in its viral genome. This concept has been exhibited by the attenuated herpes simplex virus type 1 (HSV-1), termed talimogene laherparepvec (Imlygic or T-VEC) which has been modified to produce the cytokine granulocyte–macrophage colony-stimulating factor (GM-CSF) to enhance anti-tumor immunity for the treatment of advanced melanoma (Andtbacka et al., 2015; Puzanov et al., 2016). When GBM tumor cells are lysed during treatment with the engineered virus, the tumor-associated antigens released will serve as neoantigens, which are antigens that were previously not presented to the immune system. These antigens are processed and presented by dendritic cells which can be activated by GM-CSF. This enables the priming of *de novo* anti-tumor CD8^+^ T cell responses toward previously unrecognized antigens. Notably, an oncolytic HSV expressing, IL-12, a potent anti-tumor and anti-angiogenic cytokine has shown to decrease neovasculature and tumor T regs (T regulatory cells) as well as induce T cell-associated immunity in an immunocompetent GSC model (Cheema et al., 2013). ZIKV can be used in gene therapy to deliver such immunostimulatory proteins which have therapeutic effects on uninfected cancer cells. Nevertheless, the application of ZIKV as gene delivery system may need to be further investigated to determine its efficacy. OV can act as a vaccine by expressing tumor-associated antigens or foreign antigens in the tumor which helps the immune system to recognize and target cancer cells expressing these antigens. The combination of OV-mediated cell lysis and tumor-associated antigen expression in the tumor leads to enhanced T-cell activation and migration (Diaz et al., 2007). The tumor-selective adaptive immunity would help to decrease tumor recurrence and metastases as well as improve the patient’s outcome.

Combining OV therapy with other types of treatments such as immunotherapy, biotherapy, chemotherapy, and/or radiotherapy was shown to enhance anticancer effect. Thus, combining the use of ZIKV with other types of therapy may help to target DGCs and distant metastases. Although attenuated ZIKV has been found to have additive effects when combined with temozolomide chemotherapy (Zhu et al., 2017), the detailed mechanism of the treatment remains to be elucidated. The understanding of interactive effect between OV and chemotherapy agents is necessary to best optimize the treatment combination for maximizing patient survival as the treatments’ mechanisms of action may counter the effect of one another. For instance, the use of cytostatic agents like taxanes damages microtubules that are required for adenovirus replication and decreases the efficacy of the OV when used simultaneously or before the virus administration (Yu and Fang, 2007). Thus, the use of different agents at different time order may cause different efficacy. Besides that, the expression level of proteins which are utilized by ZIKV for viral entry and replication such as AXL and MSI1 in tumor cells may affect the efficacy of the OV therapy. Hence, screening patients for expression of these proteins in tumor cells are needed to identify patients who are more likely to respond to the treatment. As such, further studies are required to identify other determinants of ZIKV infection in tumor cells. In addition, in areas where ZIKV is endemic, a large percentage of the population may be immunized against the virus. The immune system may rapidly clear the virus through antibody response before they could reach the CNS in a secondary viral exposure. Consequently, this OV strategy might only display efficacy for GBM patients who are negative for ZIKV, which may limit the use of the virus as a therapeutic agent. Hence, screening of patients may be required to better identify individuals who will benefit from the OV treatment.

## Author Contributions

VB and KS conceptualized the study, worked on the methodology, wrote, reviewed, and edited the manuscript. KS prepared the original draft of the manuscript. VB supervised the study.

## Conflict of Interest

The authors declare that the research was conducted in the absence of any commercial or financial relationships that could be construed as a potential conflict of interest.
